# A Policy Call to Address Rare Kidney Disease in Health Care Plans

**DOI:** 10.2215/CJN.0000000000000220

**Published:** 2023-06-09

**Authors:** Raymond Vanholder, Rosanna Coppo, Willem J.W. Bos, Elaine Damato, Fadi Fakhouri, Alister Humphreys, Ionut Nistor, Alberto Ortiz, Michele Pistollato, Eveline Scheres, Franz Schaefer

**Affiliations:** 1 European Kidney Health Alliance (EKHA), Brussels, Belgium; 2 Nephrology Section, Department of Internal Medicine and Pediatrics, University Hospital Ghent, Ghent, Belgium; 3 Fondazione Ricerca Molinette, Regina Margherita Hospital, Turin, Italy; 4 Department of Nephrology, Leiden University Medical Centre, Leiden, The Netherlands; 5 Department of Internal Medicine, St Antonius Ziekenhuis, Utrecht, The Netherlands; 6 Life Sciences, Charles River Associates, Mexico City, Mexico; 7 Department of Nephrology and Hypertension, Department of Medicine, Centre hospitalier universitaire Vaudois, Lausanne, Switzerland; 8 Faculty of Biology and Medicine, University of Lausanne, Lausanne, Switzerland; 9 Patient, United Kingdom; 10 Faculty of Medicine, University of Medicine and Pharmacy “Grigore T. Popa,” Iaşi, Romania; 11 Nephrology Department, Dr C.I. Parhon Hospital, Iaşi, Romania; 12 Methodological Centre for Medical Research and Evidence-Based Medicine, University of Medicine and Pharmacy “Grigore T. Popa,” Iaşi, Romania; 13 IIS-Fundación Jimenez Diaz; Professor of Medicine, Autonomous University of Madrid, Madrid, Spain; 14 Clinical Nephrology Governance, European Renal Association, Madrid, Spain; 15 Life Sciences, Charles River Associates, London, United Kingdom; 16 Division of Pediatric Nephrology, Center for Pediatrics and Adolescent Medicine, University of Heidelberg, Heidelberg, Germany

**Keywords:** CKD, health policy, policy

## Abstract

Despite a large number of people globally being affected by rare kidney diseases, research support and health care policy programs usually focus on the management of the broad spectrum of CKD without particular attention to rare causes that would require a targeted approach for proper cure. Hence, specific curative approaches for rare kidney diseases are scarce, and these diseases are not treated optimally, with implications on the patients' health and quality of life, on the cost for the health care system, and society. There is therefore a need for rare kidney diseases and their mechanisms to receive the appropriate scientific, political, and policy attention to develop specific corrective approaches. A wide range of policies are required to address the various challenges that target care for rare kidney diseases, including the need to increase awareness, improve and accelerate diagnosis, support and implement therapeutic advances, and inform the management of the diseases. In this article, we provide specific policy recommendations to address the challenges hindering the provision of targeted care for rare kidney diseases, focusing on awareness and prioritization, diagnosis, management, and therapeutic innovation. In combination, the recommendations provide a holistic approach aiming for all aspects of rare kidney disease care to improve health outcomes, reduce the economic effect, and deliver benefits to society. Greater commitment from all the key stakeholders is now needed, and a central role should be assigned to patients with rare kidney disease to partner in the design and implementation of potential solutions.

## Introduction

In 2020, targeted care was available for only 500 of the more than 8000 diagnosable rare diseases.^1^ Among CKD, rare kidney diseases (affecting <1 in 2000 people^2^) have up to now received insufficient attention, although an estimated 11.9% of adults and most children with kidney failure suffer from rare kidney diseases.^3^ This contrasts with the more common causes (*e.g.*, hypertension and diabetes).^4^ This lack of targeted approaches results in a substantial risk of developing kidney failure, with a need for KRT (dialysis or transplantation),^5^ and reduced survival and quality of life,^6^ resulting in significant personal, health care system, and societal costs.^7^

A broad array of barriers hampers the development of targeted approaches. These barriers include (*1*) general practitioners having not enough experience; (*2*) researchers missing access to large databases; (*3*) industry facing problems conducting controlled trials; and (*4*) nephrologists lacking diagnostic tools, validated biomarkers, and guidance for targeted therapeutic options. Hence, there is currently a high unmet need for innovative research and a lack of therapeutic solutions that directly target rare kidney diseases.

The aims of this policy paper are to define the obstacles to efficient health care for rare kidney diseases. In addition, it also outlines strategies for targeted policy actions and prioritization that would facilitate slowing down progression. Finally, it addresses the challenges hindering targeted care. With this text, we not only target policy makers, insurers, and funders defining care and research objectives but also providers, researchers, medical societies, and practitioners. In addition, this article should also be of use to patients, especially those in search of inspiration for advocacy.

## Methodology

This position statement builds on a literature review and the views of an expert advisory board.

**Figure 1:**
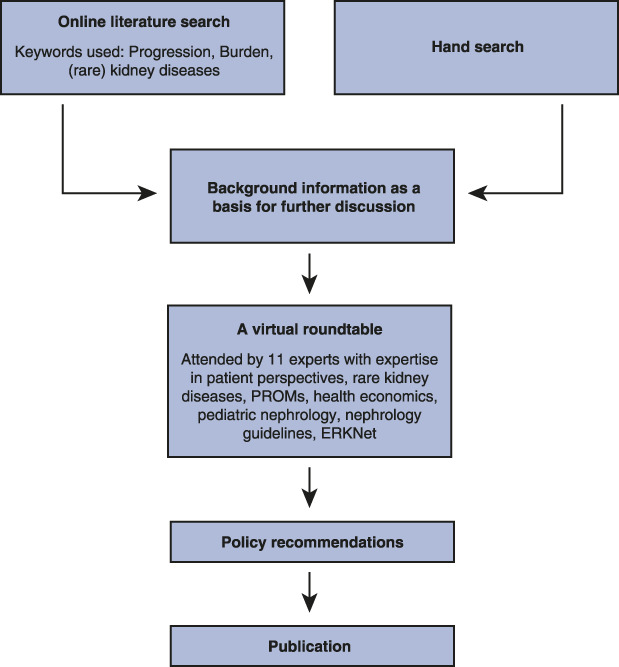
**Methodology used to develop current publication.** ERKNet, European Rare Kidney Disease Reference Network; PROMs, patient-reported outcome measures.

First, we conducted a review of the literature published after 2015 on the diagnosis and management of rare kidney diseases as well as gaps in innovative research, health care approaches, and policy actions to deal with shortcomings in research and clinical care (Figure 1). To find relevant publications, the search terms “progression,” “burden,” and “kidney diseases” or “rare kidney diseases” were used in Google Scholar and PubMed. The retrieved publications were ranked according to their relevance to the search terms and used as a basis for further discussion by the expert group together with additional articles referred to in these articles and included through hand search. Then, a virtual roundtable was organized by the European Kidney Health Alliance (EKHA—for further information about EKHA, see acknowledgments) on February 2–3, 2022. The invited experts have a variety of complementary expertise, including patients' perspectives, rare kidney diseases, patient-reported outcome measures (PROMs), health economics, nephrology guidelines, pediatric nephrology, and links to the European Rare Kidney Disease Reference Network (ERKNet). A total of 11 experts have been involved, all of whom provided their feedback.

On the basis of this search and the roundtable, a comprehensive text was distilled,^8^ which is summarized in the current publication.

## Literature Analysis

### Awareness and Prioritization

A fundamental obstacle to an efficient approach to rare kidney diseases is a lack of awareness, resulting in their nonprioritization by policy makers. CKDs at large (which include rare kidney diseases) are not listed among the health research priorities of the European Commission.^9^ In addition, at the population level, awareness is limited. Less than 10% of patients with stage 3 CKD are aware of their disease.^10,11^ At the physician level, awareness of rare kidney diseases is low, resulting in patients being referred to a nephrologist too late.^11^

### Diagnosis

Diagnosis of rare kidney diseases is problematic and has historically relied on kidney biopsies, which have shortcomings (invasive, risk of bleeding, insufficient sample size for diagnosis, limitations in access to histologic techniques and expertise for interpretation, and limited prognostic value) and are not systematically used.^12^ Patients with rare kidney diseases often spend years visiting multiple health care providers before receiving an accurate diagnosis. Next-generation sequencing techniques are emerging as the new diagnostic standard for genetic rare kidney diseases as they noninvasively amplify diagnostic accuracy, help decipher molecular mechanisms, allow genetic counseling, and offer possibilities for carrier testing.^10^ Unfortunately and despite rapidly decreasing costs, access to genetic testing is still limited in many health care systems. In addition, variants of unknown significance often make interpretation challenging. Silico models and expert consensus may help in solving part of these issues.^13^

Additional diagnostic methods also have a limited effect. Although population-wide urine screening programs are cost-effective,^14^ only 24% of countries globally deploy a CKD detection program on the basis of national guidelines or policies.^15^ Especially, for nongenetic rare kidney disorders, the identification of specific urinary biomarkers (liquid biopsy) is urgently needed.^16^

The need for adequate and timely screening is not specific to rare kidney diseases but may necessitate more than the usual determination of estimated GFR and albuminuria (hematuria, urinary sediment morphologic analysis, genetic screening, and specific biomarkers). With today's limited therapeutic resources, some may question the usefulness of an early diagnosis, but it will still allow a number of appropriate lifestyle measures and BP control. Early action will gain importance because the number of specific therapies will increase, as has already been demonstrated in selected diseases (*e.g.*, cystinosis^17^ and primary hyperoxaluria^18^).

### Lack of Innovation

There have been therapeutic advances for some conditions, such as atypical hemolytic uremic syndrome,^19^ lupus nephritis,^20^ cystinosis,^21^ membranous nephropathy,^22^ primary hyperoxaluria,^23^ and autosomal dominant polycystic kidney disease,^24^ while other approaches are under consideration.^25–27^ However, much more rare kidney diseases face therapeutic challenges, while currently accepted therapies may show differences in efficacy per individual patient, highlighting the need for more funding and investments in innovative therapies.^8^ Even if effective therapies are available, the lack of accurate biomarkers makes it difficult to predict or monitor progression, identify clinical phenotypes, and conduct clinical studies.^16^

### Management

In addition, the limited usage of clinical guidelines by clinicians,^28^ the scarcity of specific recommendations for rare kidney diseases, limited access to nephrology care and multidisciplinary teams,^29^ poor therapeutic adherence, health illiteracy,^30^ and limited interaction between patients and primary care physicians^31^ all jeopardize the proper management of rare kidney diseases.

The lack of targeted approaches places a considerable clinical and economic burden on society: As these diseases evolve to an advanced stage, they increase the risk of a large range of complications, each with an effect on health economy and outcomes, even well before KRT is needed.^32^ As CKD acts as a comorbidity accelerator,^33^ it reduces life expectancy at all stages and ages.^34^ In addition, health-related quality of life is severely affected and decreases as kidney insufficiency progresses.^35^ Children born with severe congenital and rare nephropathies face a high likelihood of altered physical, cognitive, and psychosocial development.^10^

Although transplantation is the ideal KRT,^36^ patients with rare kidney diseases may relapse after transplantation or be noneligible.^37^ For these patients, the disease burden is extremely high because dialysis with worse outcomes and quality of life is the only possible remaining option.

The potential economic effect is considerable, despite the small population size, as indicated by our estimates on the basis of the prevalence of some important rare kidney diseases (Table 1).^38–55^ This analysis essentially focuses on the cost of patients receiving dialysis therapy. It is difficult to find reliable data on the cost of these earlier CKD stages, especially regarding rare kidney diseases, but we can extrapolate from overall CKD data that the costs for CKD patients not on KRT will exceed costs for patients on KRT^6,56^ due to the much larger number of patients with early-stage CKD. Rare kidney diseases may impose higher relative costs than other causes of CKD at similar stages, either because of associated conditions (*e.g.*, other affected organs in inherited diseases) or more rapid progression, as reported for autosomal dominant polycystic kidney disease.^57,58^ To put costs of KRT into perspective compared with other health care costs, it is generally accepted that KRT consumes at least 2% of overall health care budgets for 0.1%–0.2% of the general population.^31^ Lower-income countries in general consume proportionally more of their health care budgets on KRT.^55,59^ Only if affordable new treatments emerge with the potential of preventing progression, can a reduction of disease burden and the annual cost per patient be expected.

**Table 1:** Estimated annual economic burden of dialysis in patients with rare kidney diseases

Disease	Worldwide Prevalence (×10^3^)	% with Kidney Failure	Cost LIC (×10^6^)	Cost MIC (×10^6^)	Cost HIC (×10^6^)	Global Cost (×10^6^)
Alport syndrome	157.2	51	$46.9	$808.5	$458.8	$1314.2
Atypical hemolytic uremic syndrome	39.3	65	$14.9	$257.7	$146.1	$418.7
Autosomal dominant polycystic kidney disease	12,500.0	28	$2040.9	$35,213.9	$19,981.3	$57,236.1
Focal and segmental glomerulosclerosis	552.0	50	$16.1	$277.4	$157.4	$450.9
C3 glomerulopathy	78.6	50	$23.0	$396.3	$224.9	$644.2
Goodpasture syndrome	7.9	30	$1.3	$23.8	$13.5	$38.6
IgA nephropathy	1988.6	20	$232.5	$4010.9	$2275.9	$6519.3
Membranous nephropathy	4345.0	10	$253.9	$4381.7	$2486.4	$7122.0

The estimated worldwide prevalence of each rare disease was extrapolated from refs 38–46. We applied the prevalence statistic to the populations living in low-, middle-, and high-income country regions as provided by the World Bank: https://data.worldbank.org/indicator/SP.POP.TOTL. Similarly, the percentage of patients with rare kidney diseases with kidney failure is based on refs. 45,47–54. The global annual economic burden of kidney failure patients who progressed to dialysis was calculated based on the data provided in ref. 55. These figures do not account for indirect costs (hospitalization, drugs, physiotherapy, and transport) and productivity losses, which would add considerably to the global cost. Cost calculations assume that every valid candidate would be treated by dialysis. Amounts mentioned are not actual costs but are estimated values, based in part on extrapolations. LIC, low-income countries; MIC, middle-income countries; HIC, high-income countries.

### Summary

There are still large gaps in policy and treatment of rare kidney diseases regarding awareness, prioritization, diagnosis, innovation, and management (Table 2). Health care systems have traditionally focused on managing rare kidney diseases only after their progression to advanced stages rather than investing resources in early detection and delay of progression. Despite the worrying consequences, health care plans rarely focus on determining early enough whether kidney disease is caused by a rare condition.

**Table 2:** Challenges hindering the provision of targeted care for rare kidney diseases

Challenges	Description
Awareness and prioritization challenges	• Limited prioritization by policy makers and absence of guidelines • Not enough focus on avoiding disease progression and too much focus on managing kidney failure • Limited physician and patient awareness of rare kidney diseases • Limited availability of data on rare kidney diseases • Few patient advocacy groups focused on rare kidney diseases^a^
Diagnostic challenges	• Limited implementation of urine screening programs to diagnose kidney disease • Limitations of current diagnostic methods • Lack of accurate biomarkers • Limited knowledge of underlying defects of those rare kidney diseases that are genetically determined • Delay in receiving an accurate diagnosis • Limited access to genetic testing
Research and development challenges	• Limited availability of targeted treatments • Clinical study design challenges • Poor translation of research developments into clinical care
Management challenges	• Lack of specific clinical guidelines • Limited use of existing clinical guidelines by clinicians • Limited access to nephrologist care • Poor management of kidney function decline and progression of the disease • Inequities in the level of access to minimal preventive care, hemodialysis, and transplantation, among countries and patient subgroups • Limited contact time between physicians and patients • Limited access to multidisciplinary teams

a Some patient organizations focusing on rare kidney diseases and kidney diseases at large are presented in Table 5.

## Discussion

**Figure 2:**
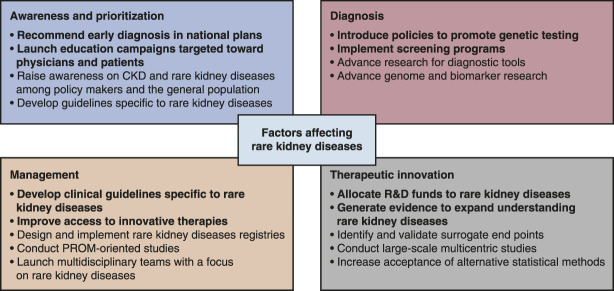
**Four categories that need policy action regarding rare kidney diseases and potential policy solutions for each.** The items in bold were prioritized by the expert panel responsible for this publication. PROM, patient-reported outcome measure; R&D, research and development.

Policy actions should be considered to increase awareness, improve diagnosis, support therapeutic advances, and inform the management of rare kidney diseases (Figure 2 and Table 3). On the basis of the obtained information and the discussion during the roundtable, the expert panel ranked the most important focus points for policy action in the order of importance (Table 4).

**Table 3:** Approaches to modify health care systems to allow efficient approaches to rare kidney diseases

Potential Policy Approaches to Address	Action Points
Awareness and prioritization	• Increase awareness of the clinical and economic burden of rare kidney diseases • Generate evidence to guide the development of national CKD plans and guidelines for rare kidney diseases • Introduce targeted measures to increase physicians' awareness of available screening programs • Support the organization of patient registries and initiatives to extend or combine databases • Introduce educational campaigns targeted to the general public • Increase focus on early detection and preventive measures and recommend inclusion of preventive measures in health care action plans
Diagnostic	• Collaborate to advance research on rare kidney disease diagnosis • Expand genetic testing offerings • Implement or expand urine screening programs
Research and development	• Allocate funds for research and development of novel diagnostic tools and treatments for rare kidney diseases • Increase efforts to translate research developments into clinical care • Establish national and international initiatives to generate evidence on rare kidney diseases, if needed based on surrogate end points (*e.g.*, in too small populations for controlled hard end point studies) • Encourage stakeholders to invest in and collaborate on the inclusion of PROMs as a research target
Management	• Develop clinical guidelines specific for the management of rare kidney diseases • Introduce initiatives to homogenize access among countries and social classes to preventive care, hemodialysis, and transplantation • Incentivize medical graduates to specialize in nephrology • Establish multidisciplinary teams to better manage rare kidney disease patients

PROMs, patient-reported outcome measures.

**Table 4:** Most important elements to be considered for policy action as defined by the expert panel participating in this project, with the corresponding barriers and involved stakeholders

Policy Action	Barriers (Lack of Solutions)	Involved Stakeholders
Recommend early diagnosis in national plans	Structured action plans, willingness to implement early screening, inefficient screening and interpretation of results	Governments, regulators, medical professionals
Increase physicians' awareness of urine screening programs and their implementation	Education, organization	Medical associations, medical professionals
Develop and expand existing clinical guidelines specific to rare kidney diseases	Evidence, training in evidence-based medicine and guideline development, funding	Medical associations, guidance bodies
Introduce policies to promote clinically relevant genetic testing	Funding; appropriate analytic technology, technical training in genetic analysis, trained personnel, targeted requests by clinicians	Governments, funders, research institutes, researchers, medical professionals
Allocate more research and development funds to rare kidney diseases	Funding, regulation	Governments, funders
Provide early optimal access to innovative treatments	Funding, evidence base, education of medical professionals, affordable price setting	Governments, pharmaceutical industry
Generate evidence to expand the understanding of rare kidney diseases	Funding, organization, privacy regulations	Pharmaceutical industry, medical professionals, research institutions, governments, funders
Set up specific rare kidney disease registries	Funding, infrastructure for big data analysis, privacy regulations, transparency in data communication and sharing	International, national, and regional medical associations; pharmaceutical industry

This ranking was the final result of the 2-day European Kidney Health Alliance Roundtable on the topic. The first ranking round was spread over five topics: (*1*) lack of awareness; (*2*) the diagnostic challenge; (*3*) the research and development challenges; (*4*) the health care system challenges; and (*5*) management challenges. The top scores for each topic were then submitted to a final vote with the intention to define which topics were considered most important for the panel.

The order from top to bottom indicates the order of importance according to the panel of experts, but this order may be adapted to the local needs of countries or regions. The ranking should also be considered as specific for this panel in the situation of this specific meeting. A different panel of experts might have come to different conclusions.

### Increase Awareness

The general perception that kidney diseases at large (including rare kidney diseases) receive less attention than other diseases cannot be denied, although it is difficult to support this statement by objective figures. One reason may be that many other rare diseases are perceived to be fatal. By contrast, many people not directly confronted with kidney diseases think that the problem of kidney failure is solved by dialysis and transplantation, without knowing that CKD is a silent killer leading to premature death in all those affected, with dialysis and even transplantation never restoring outcomes to normal. One additional problem is that people with kidney diseases are often too discreet vis-à-vis others about their condition and not always inclined to become vocal, which may be due to their multimorbid condition, combined with cognitive dysfunction, often making active advocacy discussions demanding.

To increase awareness and prioritization among policy makers, evidence to guide optimal approaches is needed. Transnational registries and other initiatives, such as public–private partnerships, can help build large databases to provide this evidence. Policy makers should be made aware of the clinical, mental, and economic burden of rare kidney diseases and the benefits of targeted approaches. Awareness among physicians and patients can be improved through comprehensive guidelines and educational campaigns.

All those involved, including especially the implicated health care professionals, but, if feasible, also the patients with CKD, should do as much effort as possible to inform the public about the risks and burden of rare kidney diseases. Patients themselves and patient organizations (Table 5) may be the most credible spokespersons to explain the burden of rare kidney diseases and should also be involved as much as possible in defining targets for innovation and optimal care. The role of patients is quintessential in awareness campaigns and in meetings with all those involved in policy decisions. More availability of information about their situation should help to create the desired level of awareness.

**Table 5:** Worldwide organizations focusing on rare kidney diseases

Organization	Acronym	Location	Main Focus
European Patient Advocacy Group within European Rare Kidney Disease Reference Network	ePAG in ERKNet	Europe	All rare kidney diseases
Federation of European Patient Groups with rare/genetic kidney diseases	FEDERG	Belgium	All rare/genetic kidney diseases
Alport Syndrome Foundation		USA	Alport syndrome
Atypical hemolytic uremic syndrome alliance	aHUS alliance	International	Atypical hemolytic uremic syndrome
Ciliopathy Alliance		Europe	Ciliopathies including polycystic kidney diseases
NephCure Kidney International		USA	Rare nephrotic syndromes
NephcEurope		Europe	Rare nephrotic syndromes
Cystinosis Network Europe		Europe	Nephropathic cystinosis
Cystinosis Research Foundation		USA	Nephropathic cystinosis
Polycystic Kidney Disease Foundation	PKD Foundation	USA	Polycystic kidney disease
Rare Kidney Disease Foundation		USA	Adult tubulointerstitial kidney disease
Association pour l’Information et la Recherche sur les Maladies rénales Génétiques France, España	AIRG. AIRG-E	France, Spain	Genetic rare kidney diseases

Regarding CKDs at large, various initiatives have been launched to increase awareness and to facilitate research and innovation such as “The Decade of the Kidney” (2020–2030),^60^ which is led by the American Association of Kidney Patients and supported by EKHA. This initiative is aimed at kidney diseases at large, which by definition also include rare kidney diseases, but it does not address them specifically. Thus, targeted initiatives are needed to specifically support rare kidney diseases.

### Improve Diagnosis

Diagnostic challenges could be addressed by greater scientific collaboration, implementation of first-line genetic testing,^61^ and identification of alarm signs through the implementation of especially urinary screening programs. Collaborative research should be encouraged to detect genetic causes of rare kidney diseases and reliable diagnostic biomarkers and predictors of progression.^62^ Further research efforts should help identify the phenotypes for whom genetic testing and targeted treatment would have added clinical value. A tentative list of rare kidney diseases where genetic disease identification is likely to change clinical practice has recently been published^61^ and needs to be validated and refined.

### Support Therapeutic Approaches

More funds for basic and translational research should be allocated, incorporating shared priorities from patients, caregivers, and health care providers.^63^ Large-scale multicentric initiatives should underpin the evidence base for effective therapies, combined with the search for validation and implementation of surrogate end points (such as improved levels of proteinuria or GFR slope) that are technically feasible on a large scale, reproducible, and acceptable for reimbursement bodies. Those would enable more controlled clinical studies in rare kidney diseases with the possibility to obviate the need for hard outcome studies, for which a large number to treat and a long follow-up time are needed before valid conclusions can be drawn. In addition, research for alternative statistical trial designs for small populations and efforts to efficiently identify candidates for clinical trials might be helpful. With more than 16,000 patients enrolled at 50 centers since 2019, the European Rare Kidney Disease Registry exemplifies how to facilitate the identification of suitable patients for trials.^64^

As PROMs ensure that interventions effectively improve the conditions that matter most to patients, there should be investment and collaboration from all stakeholders in the development of objective parameters for the assessment of PROMs, as well as PROM-oriented studies. The European Rare Disease Coordination and Support Action has recently collated an online repository of PROMs for rare diseases. Only eight of 811 PROMs addressed rare kidney diseases, demonstrating the eminent need to invest in research on PROMs for this disease domain.^65^

### Management

To inform management, the scientific community should develop clinical guidelines specific for rare kidney diseases and establish protocols targeted toward primary care and other non-nephrology physicians, providing a basis for standardized diagnoses and therapies.^10^ The units in ERKNet which adopted 40 existing guideline documents for management started a systematic effort to fill in areas where guidance was lacking. Ten guideline documents have been published to date by ERKNet specialists, usually in collaboration with other experts in adult and pediatric nephrology.^66^ Centralized multidisciplinary teams—including nephrologists and other specialties—can facilitate the uptake of these guidelines and optimize management. Referral time could be reduced by incentives encouraging medical graduates to specialize in nephrology and advance innovation by enthusing them to research rare kidney diseases.

This text contains a number of recommendations on how more attention for rare kidney diseases could be obtained. Stakeholders can make their choice among the proposed options, in function of their situation. Hopefully, this may help to generate better approaches and outcomes for this forgotten population.

By adopting a holistic approach targeting all aspects of rare kidney disease care, it should be possible to improve patient quality of life and health outcomes and reduce the economic effect of the later-stage disease. A central role should be assigned to patients with rare kidney disease patients as partners in the definition and implementation of solutions. Greater commitment from all key stakeholders is now needed to consider and eventually adopt the policy solutions identified in this study.
